# (*S*)-2-[(*S*,*Z*)-3-Bromo-1-nitro-4-phenyl­but-3-en-2-yl]cyclo­hexa­none

**DOI:** 10.1107/S1600536811025633

**Published:** 2011-07-06

**Authors:** Chao Wu, Long Zhao, Ai-Bao Xia

**Affiliations:** a State Key Laboratory Breeding Base of Green Chemistry-Synthesis Technology, Zhejiang University of Technology, Hangzhou 310014, People’s Republic of China

## Abstract

In the crystal structure of the title compound, C_16_H_18_BrNO_3_, the two stereogenic centres both have an *S* configuration. The cyclo­hexyl ring adopts a chair conformation. In the crystal, mol­ecules are linked by weak N—O⋯Br contacts [O⋯Br = 3.289 (4) Å].

## Related literature

For related structures, see: Li *et al.* (2010 ▶); Chua *et al.* (2009 ▶). For the asymmetric Michael reaction, which in principle allows for the formation of two contiguous asymmetric centers, see: Zeng & Zhong (2009 ▶); Roca-Lopez *et al.* (2010 ▶); Tsogoeva (2007 ▶); Sulzer-Mosse & Alexakis (2007 ▶); Mukherjee *et al.* (2007 ▶).
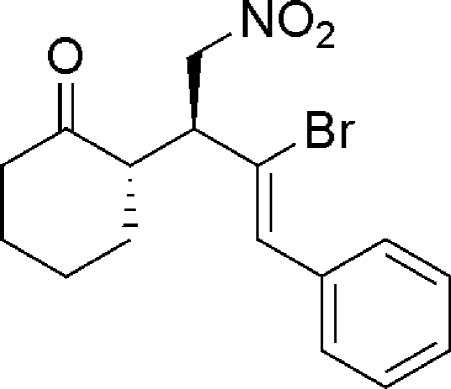


## Experimental

### 

#### Crystal data

C_16_H_18_BrNO_3_
*M* *_r_* = 352.22
Orthorhombic, 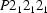

*a* = 8.0091 (3) Å
*b* = 12.2395 (6) Å
*c* = 16.3039 (7) Å
*V* = 1598.23 (12) Å^3^
*Z* = 4
Mo *K*α radiation
μ = 2.58 mm^−1^
*T* = 296 K
0.33 × 0.29 × 0.25 mm

#### Data collection

Rigaku R-AXIS RAPID/ZJUG diffractometer
Absorption correction: multi-scan (*ABSCOR*; Higashi, 1995 ▶) *T*_min_ = 0.428, *T*_max_ = 0.525
15575 measured reflections
3636 independent reflections
2369 reflections with *I* > 2σ(*I*)
*R*_int_ = 0.051

#### Refinement

*R*[*F* ^2^ > 2σ(*F* ^2^)] = 0.037
*wR*(*F* ^2^) = 0.114
*S* = 1.00
3636 reflections
191 parameters
H-atom parameters constrained
Δρ_max_ = 0.82 e Å^−3^
Δρ_min_ = −1.05 e Å^−3^
Absolute structure: Flack (1983 ▶), 1548 Friedel pairs
Flack parameter: −0.018 (17)

### 

Data collection: *PROCESS-AUTO* (Rigaku, 2006 ▶); cell refinement: *PROCESS-AUTO*; data reduction: *CrystalStructure* (Rigaku, 2007 ▶); program(s) used to solve structure: *SHELXS97* (Sheldrick, 2008 ▶); program(s) used to refine structure: *SHELXL97* (Sheldrick, 2008 ▶); molecular graphics: *ORTEP-3 for Windows* (Farrugia, 1997 ▶); software used to prepare material for publication: *WinGX* (Farrugia, 1999 ▶).

## Supplementary Material

Crystal structure: contains datablock(s) global, I. DOI: 10.1107/S1600536811025633/bt5566sup1.cif

Structure factors: contains datablock(s) I. DOI: 10.1107/S1600536811025633/bt5566Isup2.hkl

Supplementary material file. DOI: 10.1107/S1600536811025633/bt5566Isup3.cml

Additional supplementary materials:  crystallographic information; 3D view; checkCIF report

## Figures and Tables

**Fig. 1.**
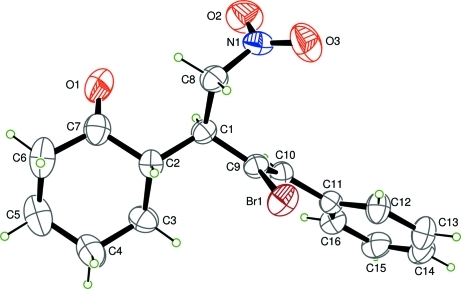
The asymmetric unit of the structure of the title compound, with the atomic labeling scheme. Displacement ellipsoids are drawn at the 50% probability level.

**Fig. 2.**
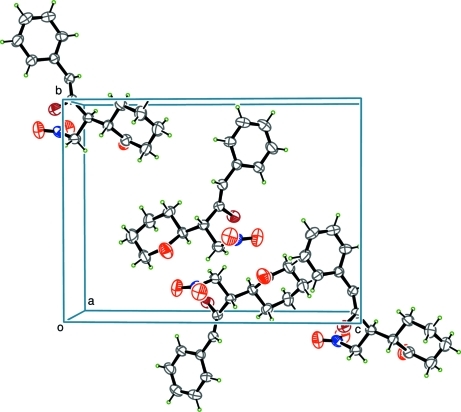
Unit cell packing of the title compound.

## supplementary crystallographic information

### Comment

Nitroalkenes are important reagents in organic chemistry and they are the most prominent Michael acceptors used in organocatalytic reactions (Tsogoeva *et al.*, 2007; Sulzer-Mosse *et al.*, 2007; Mukherjee *et al.*, 2007). Consequently, the title compound was synthesized as one of a series of solvent-free Michael products under investigation. In this paper, its absolute configuration and crystal structure are presented.The title compound is shown in Fig. 1. The cyclohexyl ring adopts a chair conformation. The plane of the phenyl ring and the least-square plane of the cyclohexyl moiety enclose an angle of 63.96 (3)°. The torsion angle  O1—C7—Br1—C9 is 139.74 (2)° The molecules are linked by weak N1—O2···Br1 contacts. The O···Br distance is 3.289 Å.

### Experimental

A mixture of (2-bromo-4-nitrobuta-1,3-dienyl)benzene (1 mmol) and cyclohexanone (8 mmol) in the presence of (*S*)-2-(pyrrolidin-2-ylmethylthio)pyridine(0.2 mmol) as amine catalyst and 4-(trifluoromethyl)benzoic acid(0.2 mmol) as cocatalyst at room temperature with stirring. After completion of the reaction, the mixture was extracted with ethyl acetate. The solvent was removed under reduced pressure and the residue was purified by silica gel column chromatography (eluent: petroleum ether-aether). Suitable crystals were obtained by slow evaporation of an ethyl ether solution.

### Refinement

H atoms were placed in calculated position with C—H ranging from 0.93 Å to 0.98 Å and refined using a riding model with *U*_iso_(H)=1.2*U*_eq_ of the carrier atoms.

### Crystal data

**Table:**

C_16_H_18_BrNO_3_	*F*(000) = 720
*M**_r_* = 352.22	*D*_x_ = 1.464 Mg m^−^^3^
Orthorhombic, *P*2_1_2_1_2_1_	Mo *K*α radiation, λ = 0.71073 Å
Hall symbol: P 2ac 2ab	Cell parameters from 10471 reflections
*a* = 8.0091 (3) Å	θ = 3.0–27.4°
*b* = 12.2395 (6) Å	µ = 2.58 mm^−^^1^
*c* = 16.3039 (7) Å	*T* = 296 K
*V* = 1598.23 (12) Å^3^	Chunk, colorless
*Z* = 4	0.33 × 0.29 × 0.25 mm

### Data collection

**Table:**

Rigaku R-AXIS RAPID/ZJUG diffractometer	3636 independent reflections
Radiation source: rolling anode	2369 reflections with *I* > 2σ(*I*)
graphite	*R*_int_ = 0.051
Detector resolution: 10.00 pixels mm^-1^	θ_max_ = 27.4°, θ_min_ = 3.0°
ω scans	*h* = −10→10
Absorption correction: multi-scan (*ABSCOR*; Higashi, 1995)	*k* = −15→15
*T*_min_ = 0.428, *T*_max_ = 0.525	*l* = −20→21
15575 measured reflections	

### Refinement

**Table:**

Refinement on *F*^2^	Hydrogen site location: inferred from neighbouring sites
Least-squares matrix: full	H-atom parameters constrained
*R*[*F*^2^ > 2σ(*F*^2^)] = 0.037	*w* = 1/[σ^2^(*F*_o_^2^) + (0.*P*)^2^ + 4.1524*P*] where *P* = (*F*_o_^2^ + 2*F*_c_^2^)/3
*wR*(*F*^2^) = 0.114	(Δ/σ)_max_ = 0.001
*S* = 1.00	Δρ_max_ = 0.82 e Å^−^^3^
3636 reflections	Δρ_min_ = −1.05 e Å^−^^3^
191 parameters	Extinction correction: *SHELXL97* (Sheldrick, 2008), Fc^*^=kFc[1+0.001xFc^2^λ^3^/sin(2θ)]^-1/4^
0 restraints	Extinction coefficient: 0.0310 (13)
Primary atom site location: structure-invariant direct methods	Absolute structure: Flack (1983), 1548 Friedel pairs
Secondary atom site location: difference Fourier map	Flack parameter: −0.018 (17)

### Special details

**Table:**

Geometry. All e.s.d.'s (except the e.s.d. in the dihedral angle between two l.s. planes) are estimated using the full covariance matrix. The cell e.s.d.'s are taken into account individually in the estimation of e.s.d.'s in distances, angles and torsion angles; correlations between e.s.d.'s in cell parameters are only used when they are defined by crystal symmetry. An approximate (isotropic) treatment of cell e.s.d.'s is used for estimating e.s.d.'s involving l.s. planes.
Refinement. Refinement of *F*^2^ against ALL reflections. The weighted *R*-factor *wR* and goodness of fit *S* are based on *F*^2^, conventional *R*-factors *R* are based on *F*, with *F* set to zero for negative *F*^2^. The threshold expression of *F*^2^ > σ(*F*^2^) is used only for calculating *R*-factors(gt) *etc*. and is not relevant to the choice of reflections for refinement. *R*-factors based on *F*^2^ are statistically about twice as large as those based on *F*, and *R*- factors based on ALL data will be even larger.

### Fractional atomic coordinates and isotropic or equivalent isotropic displacement parameters (Å^2^)

**Table:**

	*x*	*y*	*z*	*U*_iso_*/*U*_eq_	
Br1	0.63058 (7)	0.46482 (5)	0.55564 (4)	0.0619 (2)	
N1	0.1663 (6)	0.3677 (4)	0.5794 (3)	0.0571 (13)	
O2	0.0229 (5)	0.3856 (5)	0.5610 (4)	0.0905 (15)	
O1	0.1693 (7)	0.3171 (5)	0.3392 (3)	0.0885 (16)	
C11	0.4279 (6)	0.7079 (4)	0.5742 (3)	0.0460 (13)	
C2	0.4282 (7)	0.3931 (5)	0.3846 (3)	0.0495 (13)	
H2	0.5210	0.3467	0.4025	0.059*	
C1	0.3329 (6)	0.4313 (4)	0.4610 (3)	0.0466 (13)	
H1	0.2289	0.4650	0.4420	0.056*	
C10	0.3654 (7)	0.6156 (4)	0.5251 (3)	0.0462 (12)	
H10	0.2658	0.6305	0.4979	0.055*	
O3	0.2193 (7)	0.3763 (5)	0.6486 (3)	0.0933 (17)	
C15	0.4339 (8)	0.9025 (5)	0.5950 (4)	0.0655 (18)	
H15	0.4060	0.9722	0.5771	0.079*	
C16	0.3855 (7)	0.8127 (4)	0.5505 (4)	0.0536 (12)	
H16	0.3225	0.8227	0.5031	0.064*	
C14	0.5235 (8)	0.8893 (6)	0.6660 (4)	0.0655 (18)	
H14	0.5559	0.9500	0.6964	0.079*	
C3	0.5005 (8)	0.4881 (5)	0.3337 (4)	0.0604 (16)	
H3A	0.4103	0.5348	0.3152	0.073*	
H3B	0.5743	0.5314	0.3678	0.073*	
C9	0.4228 (6)	0.5152 (4)	0.5117 (3)	0.0460 (12)	
C7	0.3176 (9)	0.3261 (5)	0.3272 (4)	0.0614 (17)	
C12	0.5181 (8)	0.6956 (5)	0.6466 (4)	0.0606 (16)	
H12	0.5470	0.6260	0.6647	0.073*	
C8	0.2845 (8)	0.3328 (5)	0.5139 (4)	0.0559 (15)	
H8A	0.2324	0.2774	0.4799	0.067*	
H8B	0.3837	0.3014	0.5386	0.067*	
C6	0.4045 (10)	0.2823 (6)	0.2531 (4)	0.078 (2)	
H6A	0.3246	0.2449	0.2183	0.093*	
H6B	0.4887	0.2298	0.2698	0.093*	
C4	0.5965 (9)	0.4456 (6)	0.2599 (4)	0.079 (2)	
H4A	0.6393	0.5069	0.2286	0.094*	
H4B	0.6911	0.4028	0.2787	0.094*	
C13	0.5651 (9)	0.7863 (6)	0.6919 (4)	0.0696 (19)	
H13	0.6254	0.7772	0.7402	0.084*	
C5	0.4875 (11)	0.3752 (7)	0.2046 (4)	0.089 (3)	
H5A	0.5553	0.3445	0.1611	0.107*	
H5B	0.4019	0.4204	0.1797	0.107*	

### Atomic displacement parameters (Å^2^)

**Table:**

	*U*^11^	*U*^22^	*U*^33^	*U*^12^	*U*^13^	*U*^23^
Br1	0.0516 (3)	0.0585 (3)	0.0756 (4)	0.0081 (3)	−0.0139 (3)	−0.0041 (3)
N1	0.055 (3)	0.056 (3)	0.060 (3)	−0.008 (2)	0.007 (2)	0.013 (2)
O2	0.052 (3)	0.117 (4)	0.102 (4)	0.005 (3)	0.001 (3)	0.006 (4)
O1	0.084 (4)	0.101 (4)	0.080 (3)	−0.019 (3)	−0.015 (3)	−0.023 (3)
C11	0.046 (3)	0.047 (3)	0.045 (3)	−0.001 (2)	0.002 (2)	0.000 (2)
C2	0.053 (3)	0.050 (3)	0.046 (3)	0.005 (3)	−0.004 (2)	−0.004 (2)
C1	0.049 (3)	0.040 (3)	0.052 (3)	−0.002 (2)	−0.004 (2)	0.001 (2)
C10	0.044 (3)	0.048 (3)	0.046 (3)	0.002 (3)	−0.002 (3)	−0.002 (2)
O3	0.098 (4)	0.127 (5)	0.055 (3)	0.012 (3)	−0.002 (3)	0.010 (3)
C15	0.064 (4)	0.043 (3)	0.089 (5)	−0.001 (3)	0.006 (3)	−0.010 (3)
C16	0.053 (3)	0.042 (3)	0.066 (3)	0.006 (3)	−0.001 (3)	−0.001 (3)
C14	0.058 (4)	0.060 (4)	0.078 (5)	−0.012 (3)	0.007 (3)	−0.023 (4)
C3	0.064 (4)	0.057 (4)	0.061 (3)	0.000 (3)	0.008 (3)	0.009 (3)
C9	0.046 (3)	0.043 (3)	0.049 (3)	−0.001 (2)	0.000 (2)	0.002 (2)
C7	0.073 (4)	0.057 (4)	0.054 (4)	0.006 (3)	−0.008 (3)	−0.001 (3)
C12	0.076 (4)	0.050 (4)	0.055 (4)	0.006 (3)	−0.009 (3)	−0.008 (3)
C8	0.063 (4)	0.043 (3)	0.061 (4)	−0.003 (3)	0.005 (3)	−0.001 (3)
C6	0.100 (6)	0.073 (5)	0.060 (4)	0.021 (4)	−0.023 (4)	−0.018 (4)
C4	0.093 (5)	0.087 (5)	0.056 (4)	0.003 (5)	0.015 (4)	0.000 (4)
C13	0.082 (5)	0.065 (4)	0.062 (4)	0.000 (4)	−0.012 (3)	−0.018 (3)
C5	0.123 (7)	0.087 (6)	0.057 (4)	0.024 (5)	0.006 (4)	−0.005 (4)

### Geometric parameters (Å, °)

**Table:**

Br1—C9	1.914 (5)	C16—H16	0.9300
N1—O2	1.208 (6)	C14—C13	1.371 (9)
N1—O3	1.209 (6)	C14—H14	0.9300
N1—C8	1.490 (7)	C3—C4	1.520 (8)
O1—C7	1.209 (8)	C3—H3A	0.9700
C11—C16	1.382 (7)	C3—H3B	0.9700
C11—C12	1.393 (8)	C7—C6	1.493 (9)
C11—C10	1.472 (7)	C12—C13	1.385 (8)
C2—C7	1.528 (8)	C12—H12	0.9300
C2—C1	1.535 (7)	C8—H8A	0.9700
C2—C3	1.542 (8)	C8—H8B	0.9700
C2—H2	0.9800	C6—C5	1.536 (9)
C1—C9	1.502 (7)	C6—H6A	0.9700
C1—C8	1.532 (7)	C6—H6B	0.9700
C1—H1	0.9800	C4—C5	1.521 (9)
C10—C9	1.330 (7)	C4—H4A	0.9700
C10—H10	0.9300	C4—H4B	0.9700
C15—C14	1.371 (9)	C13—H13	0.9300
C15—C16	1.373 (8)	C5—H5A	0.9700
C15—H15	0.9300	C5—H5B	0.9700
			
O2—N1—O3	123.4 (6)	C10—C9—C1	123.8 (5)
O2—N1—C8	118.5 (6)	C10—C9—Br1	122.5 (4)
O3—N1—C8	118.1 (5)	C1—C9—Br1	113.7 (4)
C16—C11—C12	117.7 (5)	O1—C7—C6	123.8 (6)
C16—C11—C10	118.4 (5)	O1—C7—C2	121.3 (6)
C12—C11—C10	123.7 (5)	C6—C7—C2	114.7 (6)
C7—C2—C1	111.9 (5)	C13—C12—C11	120.4 (6)
C7—C2—C3	107.0 (5)	C13—C12—H12	119.8
C1—C2—C3	113.2 (5)	C11—C12—H12	119.8
C7—C2—H2	108.2	N1—C8—C1	109.8 (5)
C1—C2—H2	108.2	N1—C8—H8A	109.7
C3—C2—H2	108.2	C1—C8—H8A	109.7
C9—C1—C8	110.5 (4)	N1—C8—H8B	109.7
C9—C1—C2	114.6 (4)	C1—C8—H8B	109.7
C8—C1—C2	110.0 (4)	H8A—C8—H8B	108.2
C9—C1—H1	107.1	C7—C6—C5	110.6 (6)
C8—C1—H1	107.1	C7—C6—H6A	109.5
C2—C1—H1	107.1	C5—C6—H6A	109.5
C9—C10—C11	132.8 (5)	C7—C6—H6B	109.5
C9—C10—H10	113.6	C5—C6—H6B	109.5
C11—C10—H10	113.6	H6A—C6—H6B	108.1
C14—C15—C16	120.0 (6)	C5—C4—C3	111.8 (6)
C14—C15—H15	120.0	C5—C4—H4A	109.3
C16—C15—H15	120.0	C3—C4—H4A	109.3
C15—C16—C11	121.7 (6)	C5—C4—H4B	109.3
C15—C16—H16	119.2	C3—C4—H4B	109.3
C11—C16—H16	119.2	H4A—C4—H4B	107.9
C15—C14—C13	119.7 (6)	C14—C13—C12	120.5 (6)
C15—C14—H14	120.2	C14—C13—H13	119.8
C13—C14—H14	120.2	C12—C13—H13	119.8
C4—C3—C2	111.0 (5)	C4—C5—C6	111.3 (6)
C4—C3—H3A	109.4	C4—C5—H5A	109.4
C2—C3—H3A	109.4	C6—C5—H5A	109.4
C4—C3—H3B	109.4	C4—C5—H5B	109.4
C2—C3—H3B	109.4	C6—C5—H5B	109.4
H3A—C3—H3B	108.0	H5A—C5—H5B	108.0
			
C7—C2—C1—C9	−168.6 (5)	C1—C2—C7—O1	7.4 (9)
C3—C2—C1—C9	−47.6 (6)	C3—C2—C7—O1	−117.2 (7)
C7—C2—C1—C8	66.2 (6)	C1—C2—C7—C6	−177.6 (5)
C3—C2—C1—C8	−172.8 (5)	C3—C2—C7—C6	57.8 (7)
C16—C11—C10—C9	152.7 (6)	C16—C11—C12—C13	−1.0 (9)
C12—C11—C10—C9	−31.9 (9)	C10—C11—C12—C13	−176.5 (6)
C14—C15—C16—C11	−1.5 (9)	O2—N1—C8—C1	75.7 (7)
C12—C11—C16—C15	1.8 (9)	O3—N1—C8—C1	−104.4 (6)
C10—C11—C16—C15	177.5 (5)	C9—C1—C8—N1	62.7 (6)
C16—C15—C14—C13	0.4 (10)	C2—C1—C8—N1	−169.7 (5)
C7—C2—C3—C4	−57.4 (7)	O1—C7—C6—C5	119.3 (8)
C1—C2—C3—C4	178.8 (5)	C2—C7—C6—C5	−55.6 (8)
C11—C10—C9—C1	177.3 (5)	C2—C3—C4—C5	58.2 (8)
C11—C10—C9—Br1	−3.0 (9)	C15—C14—C13—C12	0.4 (10)
C8—C1—C9—C10	−116.9 (6)	C11—C12—C13—C14	0.0 (10)
C2—C1—C9—C10	118.1 (6)	C3—C4—C5—C6	−54.1 (8)
C8—C1—C9—Br1	63.3 (5)	C7—C6—C5—C4	51.6 (9)
C2—C1—C9—Br1	−61.7 (5)		
